# Hydrothermal Aging Properties of Three Typical Bamboo Engineering Composites

**DOI:** 10.3390/ma12091450

**Published:** 2019-05-05

**Authors:** Haiying Zhou, Ge Wang, Linbi Chen, Zhiming Yu, Lee M. Smith, Fuming Chen

**Affiliations:** 1 Key Laboratory of Bamboo and Rattan Science and Technology of the State Forestry Administration, Department of Bio-materials, International Centre for Bamboo and Rattan, Beijing 100102, China; 15288423522@163.com; 2 College of Materials Science and Technology, Beijing Forestry University, Beijing 100083, China; rmb1415926@163.com (L.C.); yuzhiming@bjfu.edu.cn (Z.Y.); 3 Mechanical and Energy Engineering, University of North Texas, Denton, TX 76203-1277, USA; LeeSmith@my.unt.edu

**Keywords:** bamboo engineering composites, hydrothermal-aging performance, thickness swelling model, Fick’s second law, MOR degradation

## Abstract

The objective of this study was to investigate the hygroscopic characteristics of three typical bamboo engineering composites (Bamboo scrimber (BS), bamboo bundle/wood laminated veneer lumber (BLVL), and bamboo laminated timber (BLT)) as well as predict their performance changes and service life in hot humid environments. The composites were subjected to three treatment conditions (23 °C, 63 °C, and 100 °C) for this experiment. The hygroscopic thickness swelling model and Fick’s second law were used to quantify the characterization and prediction of the water absorption, thickness swelling rate, and water absorption rate of BS, BLVL, and BLT. The results indicated that the order of the hygroscopic thickness swelling coefficient *K_SR_* and the diffusion coefficient D was BLT > BLVL > BS (at 23 °C and 63 °C). The optimal dimensional stability was displayed by BS, followed by BLVL and BLT. In addition to the hygroscopic properties, elastic modulus degradation was investigated. It was observed that the elastic modulus (MOR) degradation had a linear relationship with the aging temperature. After 152 h of the hydrothermal aging test (63 °C), the MOE of BS, BLVL, and BLT degraded by 44.33%, 53.89%, and 25.83%, respectively.

## 1. Introduction

Bamboo is one of the most prominent renewable biomass resources in the world. The bamboo-engineering composites represented in this study are bamboo scrimber (BS), bamboo laminated timber (BLT), and bamboo bundle/wood veneer lumber (BLVL) which are favored by consumers in China where they are widely used in construction, outdoor flooring, furniture, and transportation [1,2,3]. As a biomass resource, bamboo contains various tiny voids that make up its pore structure and its chemical composition which contains a large number of polar hydrophilic groups such as hydroxyl groups and amino groups [4,5,6], which will cause it to absorb moisture from the ambient environment. With regard to the biomass composites, the difference in damp heat sensitivity between pores, fibers, and resin matrix cause swelling in the matrix and fiber, which generates internal stress and the growth of microcracks along the interface, leading to dimensional instability and mechanical property degradation of the composites [7,8,9,10]. Therefore, it is essential to examine the hygroscopic properties of bamboo engineering composites in a hydrothermal environment and understand their performance changes.

At present, there are a few studies on the hygrothermal properties of bamboo composites. Since bamboo is a viscoelastic porous material, the pressure and temperature that it is subjected to during the preparation process cause internal stress in the bamboo composites [11,12]. As bamboo is compressed to a certain thickness, the internal stress will increase and absorb moisture under hygrothermal conditions, which will cause thickness swelling [13]. The hygroscopic thickness swelling of bamboo or wood composites is an important factor in evaluating their quality. At present, there are many studies on the hygroscopic thickness expansion of wood-based panels and wood-plastic composites, mainly focusing on the influence of composition [14,15] and structure [16,17] on thickness swelling. There are few targeted studies on the hygroscopic thickness expansion of bamboo composites. Using models to predict the thickness swelling of materials can save time and resources and is the future development trend. Shi and Gardner considered that the moisture absorption thickness swelling rate is related to the initial thickness of the board, which was used to establish the moisture absorption thickness prediction model of wood fiberboard and wood fiber/polymer composites under different temperature and humidity conditions [18]. It was found that the influence of temperature on the hygroscopic thickness swelling is significant. However, the prediction model has large errors under high-temperature conditions. Later, some scholars used the hygroscopic thickness swelling model developed by Shi and Gardner to study the thickness swelling process of different wood-plastic composites (WPC). It was found that the model can predict the hygroscopic thickness swelling process of wood-plastic composites [19,20,21,22]. These studies show that the hygroscopic thickness swelling model has certain feasibility for predicting the thickness swelling of composites containing natural fibers in a hygrothermal environment. 

The hygroscopicity of bamboo or wood materials is generally evaluated by the water absorption rate. A simple comparison of the water absorption rate does not explain the diffusion and penetration of moisture in plant fiber composites. Especially under the action of temperature, the penetration and diffusion behavior of moisture in plant fiber composites is more complicated. Using models to study the hygroscopic properties of plant fiber composites may be a method. There have been many studies on the hygroscopic properties of composites using diffusion models. Scholars in the biomass composites field introduced Fick’s second law to explain the hygroscopic mechanism of natural fiber composites. It is believed that Fick’s second law can explain the diffusion behavior of water molecules in natural fiber-reinforced composites to some extent [23,24,25,26,27]. The modulus of elasticity (MOE) of natural fiber composites is approximately inversely proportionate to its water absorption, so in the process of moisture absorption, the mechanical properties are attenuated along with the changes in weight and size. Li established an empirical 3-D model to evaluate the effect of the surrounding environment on the mechanical properties and degradation behavior of BLVL, which describes the relationship between MOE, water absorption, and aging temperature of BLVL [9]. The water absorption, thickness swelling, and mechanical performance are related to the water absorption rate of the composite. It is very important to establish a correlation model of the dimensional stability, mechanical performance, and the water absorption rate in order to study the hydrothermal aging properties of the composites.

In this study, the behavior of three types of bamboo engineering composites (BS, BLVL, and BLT) was studied under different hygrothermal conditions. The objectives of this study were: (1) to investigate the impact of different temperature and hydrothermal environments on the moisture absorption behavior of bamboo composites where the hygroscopic characteristics and the thickness swelling of the bamboo composites were investigated and predicted. From this impact the mechanical performance of the degradation mechanism of bamboo composites was analyzed in a hydrothermal environment; and (2) to develop a 3D empirical model for predicting the dimensional stability and mechanical performance of bamboo composites undergoing hygrothermal aging treatments.

## 2. Materials and Methods 

### 2.1. Bamboo Scrimber (BS)

The bamboo scrimber (BS) used was produced by Fujian Youzhu Technology Co., Ltd. (Yong’an, China) The bamboo bundle fiber used for the BS was made from Moso bamboo (Phyllostachys edulis) with an age greater than four years from Yongan, Fujian Province. The BS properties were a density of 1.25 g/cm^3^, a moisture content of 9–13%, and a thickness of 12 mm. A liquid phenol-formaldehyde (PF) resin (Taier Corporation, Guangdong, China) with 25% solid content was used as the adhesive for the bamboo bundle fiber, and its viscosity was 359.7 CP.s. (temperature of 17 °C). The hot pressing parameters were a pressing temperature of 150 °C, time of 1.5 mm/min, and hot pressing pressure of 4 MPa [28].

### 2.2. Bamboo Bundle/Wood Laminated Veneer Lumber (BLVL)

Bamboo bundle/wood veneer lumber (BLVL) is a composite of bamboo bundle veneer and eucalyptus veneer, which can exploit the advantages of the high strength and toughness of bamboo and the high yield of artificial wood. The bamboo bundle fiber was produced in Xiaotao Town, Yongan, Fujian Province. The eucalyptus board with a moisture content of about 8% and a density of 0.38–0.4 g/cm^3^ was purchased from Fujian Changzhu Industry Co., Ltd. (Yong’an, China) The bamboo bundle was impregnated with 25% PF resin and which had a glue content of about 18%. The eucalyptus veneers were coated on both sides by a glue machine. The glue content was controlled at 320–380 g/m^2^. The density and the thickness of the BLVL were 1.05 g/cm^3^ and 10.0 mm, respectively [2].

A symmetrical lay-up sandwich structure was used for the BLVL, and the order of the layers from the outer layer to the core layer was double-layered vertical bamboo bundle fibers, single-layered horizontal-grain wood veneer, and single-layered horizontal bamboo bundle fibers. The vertical bamboo bundle fibers exploited the longitudinal strength, while the transverse wood veneers and the bamboo bundle fibers enhanced the horizontal performance and dimensional stability of the BLVL.

### 2.3. Bamboo Laminated Timber (BLT)

Bamboo Laminated timber (BLT) was produced by Fujian Youzhu Technology Co., Ltd. The BLT was made from Moso bamboo strips that had undergone secondary carbonization. They were coated with urea-formaldehyde resin (UF), and the glue content was at 300 g/m^2^. The properties of the BLT was a density of 0.65 g/cm^3^, a moisture content of 9–13%, and a thickness of 11.5 mm. The hot pressing parameters used were a hot pressing temperature of 100 °C, a hot pressing pressure of 8–12 MPa, and a hot pressing time of 8.0 min [3].

### 2.4. Aging Resistance Performance and Flexural Properties Test

BS, BLVL, and BLT were sawn into 300 mm (length) × 20 mm (width) dimensions and placed in a water bath at 23 °C, 63 °C, and 99 °C, respectively. The number of sample repeats was eight. The mass, thickness, and modulus of elasticity of the samples were measured in real time. The measurement interval was short in the first 32 h, and every 24 h after the 32 h until the mass and thickness of the samples stabilized, and the immersion was ended.

## 3. Results and Discussion

### 3.1. The Effect of Ambient Temperature on the Aging Hygroscopic Thickness Swelling Rate (TS) of the Three Types of Bamboo Composites

The hygroscopic thickness swelling of natural fiber composites is related to the initial thickness of the board. The greater the thickness, the more substances such as cellulose and hemicellulose of natural plant materials are involved in the moisture absorption and expansion. Therefore, the thickness swelling rate can be regarded as a function of the thickness variation of the material. The thickness swelling coefficient *K_SR_* was introduced to quantify the thickness swelling rate of the composites [18]. However, the thickness of natural fiber composites does not expand indefinitely in a hygroscopic environment. The thickness expansion rate during the initial moisture absorption phase is slow because the pores of the natural fiber composites can accommodate the expansion of a portion of the fiber unit, and as the thickness continues to increase, the moisture absorption time increases. Then, the expansion will continue to increase at a specific rate until the thickness expansion of most of the natural fiber units is completed to achieve a balance. The value of *K_SR_* is related to the equilibrium thickness and expansion rate of the material. The relative expansion rate of the materials decreases linearly with increasing thickness. The model can quantitatively predict the thickness swelling rate and the sensitivity of different materials to temperature. The model is expressed as follows [18]:

(1) $$TS\left(t\right)=\left(\frac{H_{\infty }}{1+\frac{H_{\infty }}{H_{0}}-1e^{-K_{SR}t}}-1\right)\times 100\%$$

In the formula, *TS(t)* is the thickness swelling rate measured at time *t*, *H*∞ is the final thickness, *H*_0_ is the initial thickness, *K_SR_* is the thickness swelling coefficient, and *t* is the moisture absorption time.

**Figure 1:**
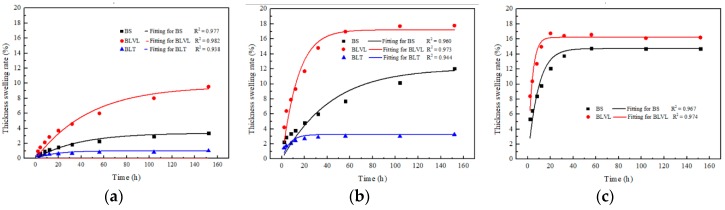
The thickness swelling rate and fitting of the three types of bamboo composites at different aging temperatures. (**a**) Aging temperature at 23 °C; (**b**) Aging temperature at 63 °C; (**c**) Aging temperature at 99 °C.

The dimensional stability of the three typical bamboo composites: BS, BLVL, and BLT in aging treatment at 23 °C, 63 °C, and 99 °C water was studied and analyzed. The variation law and the fitting curve of the thickness swelling prediction using the hygroscopic thickness swelling model are shown in Figure 1. 

Figure 1 showed that the rate of change in thickness swelling of the three bamboo composites gradually increased to an equilibrium state as the aging time extended and the higher the temperature, the greater was the thickness swelling rate. The hygroscopic thickness swelling model had a good fitting effect on the thickness swelling process, and the value of R^2^ was higher than 93%. The moisture absorption process of BS and BLVL was accurately predicted under high-temperature conditions. It indicated that the model had an accurate prediction effect on the thickness swelling of natural fiber composites with different density, structure, and composition as they were exposed to different hydrothermal environments.

**Figure 2:**
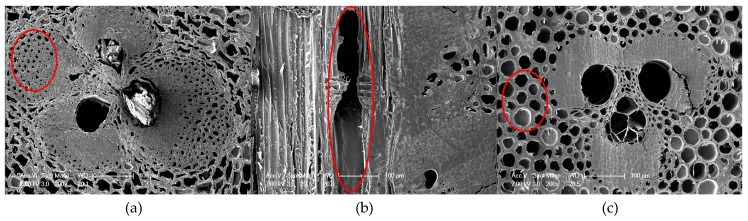
The typical structure of the electron micrographs of three types of bamboo composites at 200× magnification. (**a**) BS; (**b**) BLVL; (**c**) BLT.

The UF resin in the preparation process used on the BLT was extremely sensitive to high-temperature hydrothermal conditions, which led to structural disintegration in the 99 °C water environment. Therefore, BLT data was only recorded at 23 °C and 63 °C. Comparing the thickness swelling rate of the three bamboo composites after 152 h of hydrothermal aging treatment, it was found that the thickness swelling rate of BLVL was the largest, which was 2.83 and 9.64 times higher than BS and BLT at the 23 °C and 1.43 and 5.27 times higher at the 63 °C, respectively. In terms of components, BLVL contained wood veneers compared to BS and BLT. BLVL’s performance varied greatly in size because the wood structure possesses a lower density and a greater water absorption capacity than bamboo (Figure 2b). In the overall structure, the density of BLT was the lowest and the matrix substance combined with the water molecule was the least. Moreover, the larger parenchyma cells which were thin-walled increased the water capacity (Figure 2c). Therefore, the thickness swelling rate of BLT was the smallest. The constituent unit of BS is bamboo bundle fibers which are obtained by brooming of the bamboo, which destroyed the original gradient structure of the bamboo and greatly increased the specific surface area. This allowed the adhesive to attach to the surface of the bamboo bundle fibers more evenly, which improved the water resistance, and hence the larger density produced less void (Figure 2a), thereby increasing the dimensional stability of BS. The BS had relatively good dimensional stability and performed well under different aging conditions. 

### 3.2. The Effect of Ambient Temperature on the Hygroscopic Property of the Three Types of Bamboo Composites

**Figure 3:**
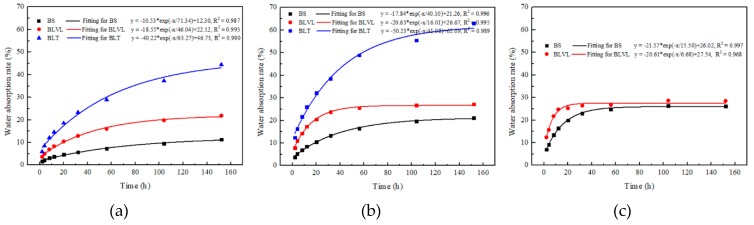
Water absorption rate and model prediction of three types of bamboo composites under different hydrothermal aging temperatures. (**a**) Aging temperature at 23 °C; (**b**) Aging temperature at 63 °C; (**c**) Aging temperature at 99 °C.

The hygroscopic curves of the three composites in water with different temperatures are shown in Figure 3. It was observed that the higher the temperature, the faster the water absorption rate (WA), and the shorter the time to reach water absorption equilibrium. The water absorption rate of BLT was the largest (23 °C and 63 °C), followed by BLVL and BS. Because BLT retained the bamboo’s vascular bundle structural characteristics, the moisture first entered the cell cavities and cell gaps of the BLT. The lower density of BLT, due to its cell cavity and cell space, caused the water absorption capacity to increase, resulting in the water absorption rate of BLT being the largest. The water absorption rate of BLVL was greater than BS due to the wood veneers in the BLVL structure.

The fitting curves of the water absorption rate of the three bamboo composites were carried out, and the fitting equation for each composite possessed a correlation coefficient greater than 95% and was obtained as follows:

*y* = A*exp(−*x*/*t*) + *y*_0_ (2)

**Figure 4:**
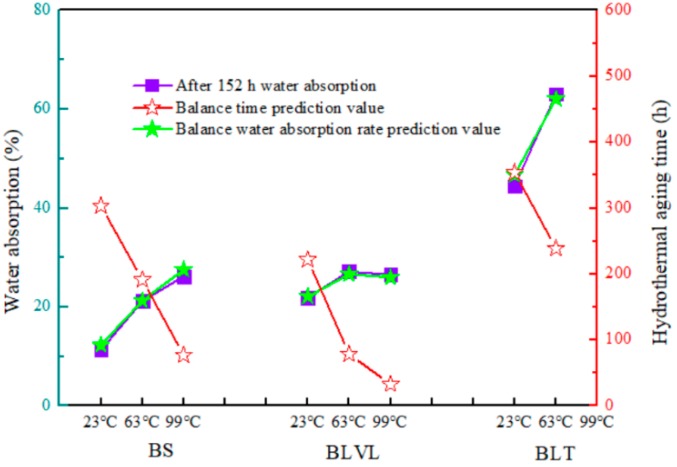
A comparison of the predicted and measured values of the three types of bamboo composites.

When the time is infinitely extended, *y_0_* in the formula is the theoretically calculated equilibrium WA. The time when WA reaches equilibrium can be calculated (WA is less than 0.1% and is regarded as the equilibrium WA). The theoretical prediction of the equilibrium time and equilibrium WA of the boards after 152 h of aging treatment are shown in Figure 3. The model of the predictive value of equilibrium WA of the three composites was slightly higher than the actual measured values. As the temperature increased, the time required to reach the equilibrium WA became shorter and shorter for the composites. According to the temperature curves (Figure 4), BLVL had the lowest sensitivity to temperature, but its WA was inferior to BS, which was consistent with the law of thickness swelling.

**Figure 5:**
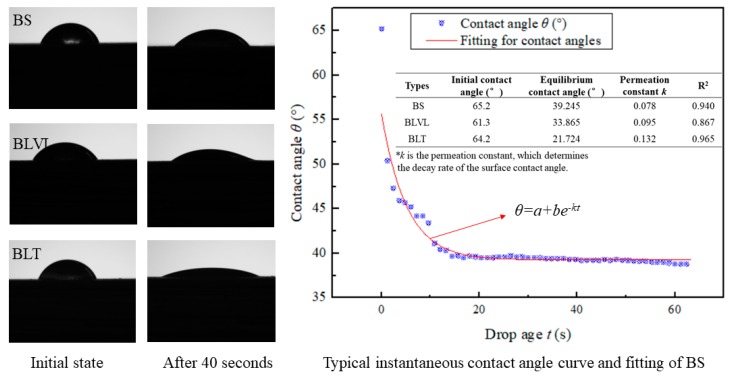
The apparent contact angles of water on the surfaces of the three bamboo composites.

The contact angles of the three bamboo composites are shown in Figure 5. The initial contact angles of BS, BLVL, and BLT were both greater than 60°, and the differences between them were small. However, it was apparent that the contact angle of the BLT changed greatly, and the contact angle of the BS did not substantially change after 40 seconds. The equilibrium contact angle and permeation constant were obtained by fitting the contact angle curves to evaluate the dynamic wetting process of the materials [29]. The permeation constants of BS, BLVL, and BLT were 0.078, 0.095, and 0.132, respectively. This indicated that BLT had the best wettability, followed by BLVL and BS. It also proved that why BLT had the highest water absorption rate. The surface contact angle is not only affected by the inherent surface free energy, but also by its surface density, texture, roughness, and other surface states. BLT had a rougher texture and a lower density (Figure 2), which were the reason for the maximum water absorption rate of BLT.

### 3.3. Simulation and Analysis of the Influence of the Aging Temperature on Dimensional Stability of the Three Types of Bamboo Composites

In order to further study the diffusion of moisture in different types of bamboo composites, Fick’s second law was introduced to quantitatively describe the hygroscopic properties. In the hygrothermal environment, the conduction mode of water molecules inside the material was in non-steady state diffusion, and the hygroscopic mechanism approximated the Fick model [30]. The Fick parameters *n* and *k*, the diffusion coefficient *D* values, and the thickness swelling coefficient *K_SR_* were obtained by the nonlinear fitting. These values were able to quantitatively characterize the dimensional stability and hygroscopic characteristics of three types of bamboo composites, as shown in Table 1. 

The effect of temperature on the hygroscopic properties of the composites was significant. It was observed that at the hydrothermal aging conditions of 23 °C (normal temperature) and 63 °C (standard temperature), the Fick coefficient *n* values of BS, BLVL, and BLT were close to 0.5, and the value of R^2^ exceeded 98%, which indicated that the three composites obeyed the Fick’ moisture absorption mechanism at lower temperatures. The *n* value of BS and BLVL were lower at 99 °C (limit temperature) and deviated from the ideal Fick’s law diffusion mechanism. The hygroscopic expansion of natural fiber composites is not only related to the hygroscopic properties of natural fibers but is also related to their thermal expansion, which means higher temperatures make the diffusion behavior of water in the bamboo composites more complicated. Therefore, the higher the temperature, the lower the accuracy of the diffusion behavior of water inside the composites based on Fick’s second law [31].

The diffusion coefficient (*D*) of the composites increases with the increase of temperature, which was consistent with the variation of the thickness swelling coefficient (*K_SR_*). Shi concluded that the thickness expansion coefficient *K_SR_* of the wood/PP fiberboard is lower than the results of this experiment [28]. Because the resin content of the wood fiber/polymer composites was up to 30%, and the resin content of the three bamboo composites prepared in the paper was 15–18%, which was only 1/2 of the former, and their natural fiber content was higher. So that the thickness expansion coefficient *K_SR_* was higher than the results of previous studies. Under the same temperature conditions, the order of the diffusion coefficient *D* was BS < BLVL < BLT, which indicated that the water had the strongest penetrative ability on BLT, followed by BLVL and BS. Because BLT maintained the original vascular bundle structure of the bamboo, the pit and pore structure of the bamboo allowed water to penetrate it the easiest. In addition, as the water was able to enter it the easiest, the density of BLT was much lower than that of BS and BLVL. The *D* value of BLVL-contained wood veneers was greater than BS, and when the temperature increased from 23 °C to 99 °C, the *D* values increased accordingly. The results indicated that the diffusion behavior of the moisture in the bamboo composites was affected by temperature. This meant the higher the temperature, the faster the movement rate and the penetration of the water molecules into the bamboo composites. The coefficient of thermal expansion of the resin and the bamboo fiber is different and the higher the temperature, the larger the difference causing internal stress and thus generating microcracks and voids, thereby increasing the water absorption capacity of the composites [27,32].

### 3.4. The Effect of Ambient Temperature on the Mechanical Properties of the Three Types of Bamboo Composites

The mechanical properties of the composites were a prerequisite for their safety and long-term use. When natural fiber composites are used, they absorb the moisture in the ambient environment. The complex diffusion behavior of water was affected by temperature, which not only caused wet deformation but also affected the mechanical properties of the composites. In order to analyze the hydrothermal aging properties of the three bamboo engineering composites at different temperatures, the attenuation law of the elastic modulus (MOE) with time was investigated. 

**Figure 6:**
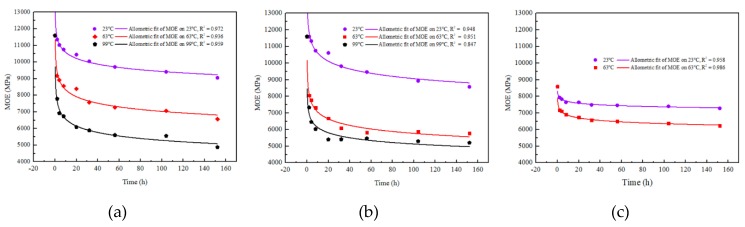

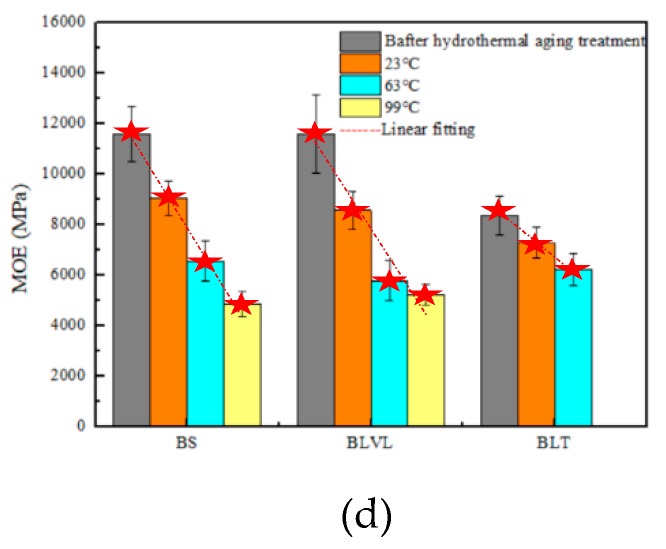
The attenuation law of the elastic modulus (MOE) of three types of bamboo composites. (**a**) BS; (**b**) BLVL; (**c**) BLT; (**d**) The comparison of MOE at different aging temperatures.

**Figure 7:**
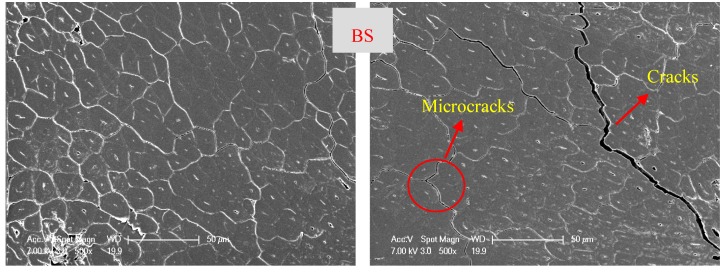

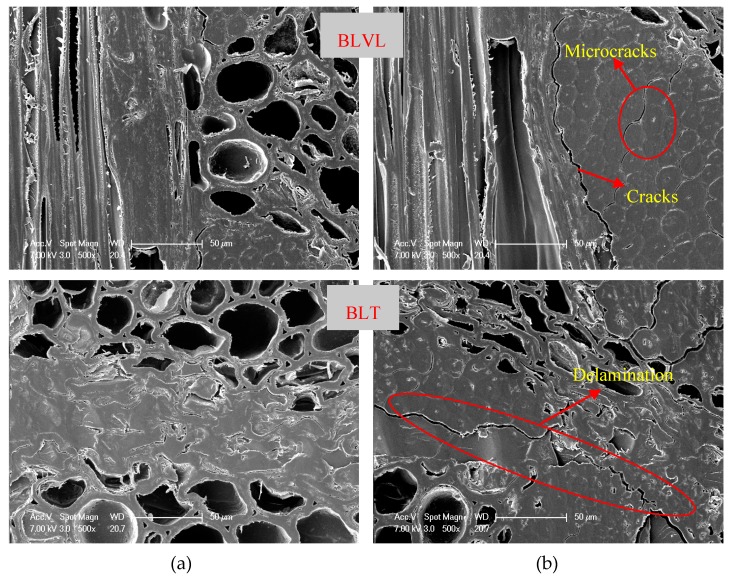
Scanning electron micrographs of the three bamboo composites. (**a**) Before the aging treatment; (**b**) After the aging treatment.

The relationship between the elastic modulus (MOE) with time for BS, BLVL, and BLT under different aging conditions is shown in Figure 6. After 152 h aging treatment, MOE of the three bamboo composites showed a tendency to decrease rapidly (in the first 20 h) and then gradually leveled off as the aging time was prolonged. Due to the failure of BLT at high-temperature conditions (99 °C), only the MOE of BS and BLVL were able to be compared at 99 °C. It was observed in Figure 6d that the MOE degradation law of the three bamboo composites was approximately linear with the temperature relationship. The degree of MOE degradation for BLVL and BS were far greater than BLT. The stiffness decay rates of BS, BLVL, and BLT after 152 h at 63 °C of aging treatment were 43.44%, 50.14%, and 25.46%, respectively. The results showed that BLT had better retention of the mechanical properties below 63 °C temperature conditions. However, the adhesive layers of the BLT were damaged under high-temperature water conditions, which indicated that BLT should be used under low temperature and humidity conditions. Under the condition of high temperature-hydrothermal coupling, the difference in sensitivity between resin and fiber led to cracks and microcracks on the surface and inside the bamboo composites, which further caused the interfacial bonding strength of the material to decrease or even delamination with the extension of time. Scanning electron micrographs of three bamboo composites before and after the aging treatment confirmed it. Figure 7 shows that the cracks and microcracks occurred in the three materials after aging for 152 h. Among them, BS and BLVL had microcracks and cracks. However, delamination occurred in BLT. Consequently, BS and BLVL had superior mechanical properties in high temperature and humidity conditions. 

### 3.5. A 3D Model Describing the Relationship between MOE, TS, and WA of BS

**Figure 8:**
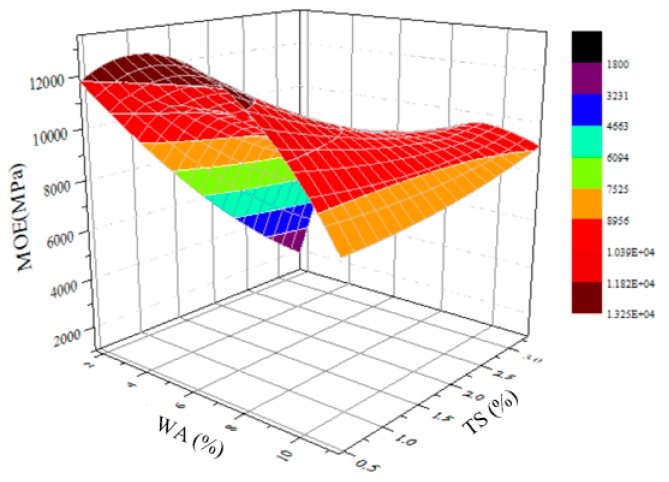
An empirical 3D model describing the relationship between MOE, TS, and WA of BS at 23 °C.

The previous analysis indicated that the mechanical strength, water absorption thickness swelling rate, and the water absorption rate of BS have a certain relationship. A 3D relationship model was established using the Origin software to describe the relationship between MOE, TS, and WA of BS under normal temperature water conditions (23 °C) (Figure 8). The attenuation of the MOE of BS increased as the water absorption rate increased and the thickness swelling rate tended to increase linearly. The result clearly showed that the combined effects of high temperature and water can lead to more severe dimensional changes and damage in the bamboo composites.

## 4. Conclusions

The effects of different aging treatments on the physical-mechanical properties of the three different types of bamboo-engineering composites were investigated. 

The hygroscopic thickness swelling models were used to predict the water absorption rate, the thickness swelling rate, and MOE of the bamboo composites. In temperatures lower than 63 °C, BLT exhibited better dimensional stability and mechanical strength retention rates, but in the case of the high-temperature hydrothermal conditions, the interface of BLT completely failed resulting in a collapse of the structural. BS possessed good dimensional stability and mechanical strength after 152 h of the hydrothermal aging test. The BLVL possessed the worst thickness swelling performance of the three composites. 

The hygroscopic thickness swelling model and Fick’s second law were used to predict the hygroscopic property of the three bamboo composites under hydrothermal conditions. The results indicated the modeling was an effective predictor of the thickness swelling and the water diffusion behavior of the composites, in different hydrothermal environments. The hygroscopic thickness swelling coefficient *K_SR_* and the diffusion coefficient *D* of BS, BLVL, and BLT were 0.025 h^−1^, 0.078 h^−1^, and 0.152 h^−1^, and 2.809 × 10^−6^ mm^2^/s, 6.486 × 10^−6^ mm^2^/s, and 34.640 × 10^−6^ mm^2^/s at 63 °C, respectively.

The elastic modulus (MOR) degradation had a linear relationship with the aging temperature. After 152 h of the hydrothermal aging test (63 °C), the MOE of BS, BLVL, and BLT degraded by 44.33%, 53.89%, and 25.83%, respectively. The combined effects of high temperature and water can lead to more severe dimensional changes and degradation of mechanical properties in the bamboo composites.

## Figures and Tables

**Table 1:** Fitting results of water absorption parameters at different aging temperatures.

Types	Temperature(°C)	Thickness Swelling Coefficient *K_SR_*(h^−1^)	R^2^	Fick’s Coefficient		R^2^	Diffusion Coefficient *D* (10^−6^)/mm^2^/s	R^2^
				*n*	*k*			
BS	23	0.027	0.998	0.443	0.067	0.999	0.760	0.999
	63	0.025	0.980	0.374	0.117	0.989	2.809	0.984
	99	0.120	0.983	0.250	0.244	0.930	6.039	0.898
BLVL	23	0.023	0.991	0.389	0.106	0.995	3.037	0.999
	63	0.078	0.987	0.443	0.034	0.999	6.486	0.895
	99	0.289	0.987	0.136	0.434	0.902	7.822	0.749
BLT	23	0.059	0.938	0.440	0.090	0.998	22.04	0.998
	63	0.152	0.972	0.355	0.148	0.993	34.64	0.982
	99	-	-	-	-	-	-	-

^1^ The case of 0 < *n* < 0.5 approximates the category of Fink diffusion [23]; *D* is the diffusion coefficient, quantitatively characterizing the permeability of the solvent minute in the material.
